# AI-supported versus manual microscopy of Kato-Katz smears for diagnosis of soil-transmitted helminth infections in a primary healthcare setting

**DOI:** 10.1038/s41598-025-07309-7

**Published:** 2025-06-27

**Authors:** Joar von Bahr, Antti Suutala, Hakan Kucukel, Harrison Kaingu, Felix Kinyua, Martin Muinde, Kevan Osundwa, Wigina Ronald, Jackson Muinde, Billy Ngasala, Mikael Lundin, Andreas Mårtensson, Nina Linder, Johan Lundin

**Affiliations:** 1 Department of Global Public Health, Karolinska Institutet, Stockholm, Sweden; 2 Institute for Molecular Medicine Finland (FIMM), University of Helsinki, Helsinki, Finland; 3 Department of Women’s and Children’s Health, Global Health & Migration Unit, Uppsala University, Uppsala, Sweden; 4 Kinondo Kwetu Hospital, Kinondo, Kwale County Kenya; 5 Department of Medical Sciences, Technical University of Mombasa, Mombasa, Kenya; 6 Ministry of Health, Kwale county, Kenya; 7 Department of Parasitology and Medical Entomology, Muhimbili University of Health and Allied Sciences, Dar es Salaam, Tanzania; 8 Department of Infectious Diseases, Uppsala University Hospital, Uppsala, Sweden

**Keywords:** Digital diagnostics, Deep learning, Neglected tropical diseases, Primary health care, Point-of-care, Whole slide imaging, Infectious diseases, Parasitic infection, Computer science, Diagnosis, Public health

## Abstract

Soil-transmitted helminths primarily comprise *Ascaris lumbricoides*, *Trichuris trichiura*, and hookworms, infecting more than 600 million people globally, particularly in underserved communities. Manual microscopy of Kato-Katz thick smears is a widely used diagnostic method in monitoring and control programs, but is time-consuming, requires on-site experts and has low sensitivity, especially for light intensity infections. In this study, portable whole-slide scanners and deep learning-based artificial intelligence (AI) were deployed in a primary healthcare setting in Kenya. Stool samples (*n* = 965) were collected from school children and Kato-Katz thick smears were digitized for AI-based detection. Light-intensity infections accounted for 96.7% of cases. Three diagnostic methods - manual microscopy, autonomous AI and human expert-verified AI - were compared to a composite reference standard, which combined expert-verified helminth eggs in physical and digital smears. Sensitivity for *A. lumbricoides*, *T. trichiura* and hookworms was 50.0%, 31.2%, and 77.8% for manual microscopy; 50.0%, 84.4%, and 87.4% for the autonomous AI; and 100%, 93.8%, and 92.2% for expert-verified AI in smears suitable for analysis (*n* = 704). Specificity exceeded 97% across all methods. The expert-verified AI had higher sensitivity than the other methods while maintaining high specificity for the detection of soil-transmitted helminths in Kato-Katz thick smears, especially in light-intensity infections.

**Supplementary Information:**

The online version contains supplementary material available at 10.1038/s41598-025-07309-7.

## Introduction

Neglected tropical diseases (NTDs) are a diverse group of conditions that receive inadequate attention in research and treatment because they primarily affect low-income countries and mainly cause chronic disability, without generating the same urgency as other global health priorities^1,2^. Soil-transmitted helminths (STHs) are the most prevalent NTDs, and in 2021, it was estimated that more than 600 million people were infected worldwide^3,4^. Children in underserved communities account for most of the morbidity caused by STHs, and infections can lead to malnutrition, impaired physical and mental development and anemia, through a complex interplay with other health determinants^3,5^. Four species account for the majority of STH infections: *Ascaris lumbricoides* (giant roundworm), *Trichuris trichiura* (whipworm), and two species of hookworm (*Necator americanus* and *Ancylostoma duodenale*)^6^.

The World Health Organization (WHO) currently recommends microscopy of stool samples prepared using the Kato-Katz technique for diagnostic tasks, such as large-scale monitoring of STH infections within mass drug administration programs and epidemiological surveys because of its simplicity, ease-of-use and ability to classify infection intensity^7,8^. The infection intensity is classified as either light, moderate, or high by quantifying parasite eggs per gram (EPG) in stool, and has clinical relevance as the intensity is correlated to the severity of symptoms^5,9^. However, limitations of microscopy of Kato-Katz smears include that an expert microscopist is required to be on-site, manual microscopy is time consuming and has low sensitivity especially for light intensity infections. The Kato-Katz technique requires the sample to be analyzed within 30–60 min, as glycerol causes disintegration of hookworm eggs^10,11^. Therefore, well trained on-site microscopists capable of performing the analysis on demand are required^9^.

Other methods have been developed to improve the diagnosis of STHs, both microscopy based methods such as formal-ethyl acetate sedimentation concentration (FLOTAC), McMaster and mini FLOTAC, and molecular methods such as polymerase chain reaction (PCR) and antigen tests^10,12^. These methods generally have higher sensitivity than Kato-Katz, but require more advanced laboratory equipment and additional technical expertise^12^. Such equipment and skills are often scarce in underserved communities where STHs are endemic; therefore, manual microscopy assessment of Kato-Katz thick smears remains the most used diagnostic method, especially in STH monitoring and control programs^7,12^. Deploying artificial intelligence (AI) supported digital microscopy for the diagnosis of STHs in Kato-Katz thick smears has been proposed as an approach to improve the diagnostic accuracy^13–15^.

Recent technological advancements have led to the development of more affordable and portable digital microscope scanners, offering a promising alternative for field-based digital diagnostics^13,14,16^. These instruments allow for digitization of entire microscope slides, i.e. whole slide imaging outside of high-end laboratories. The digitization not only facilitates remote diagnosis, quality assurance and educational reviews but also enables advanced medical image analysis using AI-based methods such as deep learning with convolutional neural networks, vision transformers, and vision-language models^13,17^.

We and others have demonstrated the potential of AI-supported digital microscopy to increase the diagnostic accuracy for STHs^14,18,19^. In our previous study we showed that AI could potentially improve the detection rate of light intensity STH infections that might be missed with manual microscopy, thus increasing sensitivity^14^. Improved sensitivity has become more important in order to achieve efficient morbidity control. The morbidity of STHs has decreased from 2.49 million daily adjusted life years in 2010 to 1.38 million in 2021, as a result of improved socio-economic standards as well as interventions with mass drug administration programs, educational efforts and improvements in water, sanitation and hygiene^3^. The global decline of STHs has led to an increased proportion of light intensity infections; therefore, more sensitive diagnostic methods are needed to ensure that decision makers are provided with robust data to guide policies on mass drug administration programs and for individual test-and-treat approaches^7,8^.

Our previous study indicated that digital whole-slide imaging combined with AI could improve the diagnosis of STHs, but indicated a need for further development of the AI and validation of the results^14^. To improve the AI-method used in the previous study, an additional deep learning (DL) algorithm to detect partially disintegrated hookworms has now been added to the original DL-algorithm, since the hookworm sensitivity was relatively low and partly disintegrated hookworm eggs were not detected by the AI in our previous study^14^. Furthermore, an AI-verificator tool is introduced to allow experts to verify AI-findings. The current study aimed to compare the diagnostic accuracy of autonomous AI, expert-verified AI and manual microscopy for STH diagnostics in a series of Kato-Katz thick smears obtained from school children in Kwale County, Kenya. The region is endemic for infections with *A. lumbricoides*, *T. trichiura*, and hookworm, but not *Schistosoma mansoni*. The three diagnostic methods were compared to a composite reference standard based on a combination of manually verified eggs in the digital and physical smears. Samples were considered positive if: (1) eggs were verified by an expert during manual microscopy or (2) two expert microscopists independently verified AI-detected eggs in the digital smears.

## Results

### Prevalence of soil-transmitted helminths

A total of 764 samples had a manual microscopy diagnosis; but 60 of those did not have an available scan. Of those 60 samples, seven (11.7%) were positive for STHs with manual microscopy: six (10%) hookworms and one (1.7%) *T. trichiura*. Of the 704 smears included in the analysis 122 (17.3%) were positive according to the composite reference standard, of which six contained mixed infections: one mixed *A. lumbricoides* and *T. trichiura* infection and five mixed *T. trichiura* and hookworm infections (Table 1).

**Table 1:** Prevalence of soil-transmitted helminths.

	Manual microscopy	Autonomous-AI	Expert-verified AI	Composite reference standard
Soil-transmitted helminths (any species)	82 (11.6%)	131 (18.6%)	129 (18.3%)	122 (17.3%)
*A. lumbricoides*	3 (0.4%)	10 (1.4%)	11 (1.6%)	6 (0.85%)
*T. trichiura*	10 (1.4%)	38 (5.4%)	35 (5.0%)	32 (4.6%)
Hookworm	70 (9.9%)	95 (13.5%)	94 (13.4%)	90 (12.8%)
Negative	622 (88.4%)	573 (81.4%)	575 (81.7%)	582 (82.6%)

Due to mixed infections, the sum of *A. lumbricoides*, *T. trichiura*, and Hookworm cases exceeds the total number of STH-positive smears.

With the additional DL-algorithm that detects disintegrated hookworm eggs, 95 smears were identified as positive for hookworms by the autonomous AI and 94 by the expert-verified AI, compared to 60 and 63, respectively, when only the original DL-algorithms were used.

### Infection intensity in positive smears

**Fig. 1:**
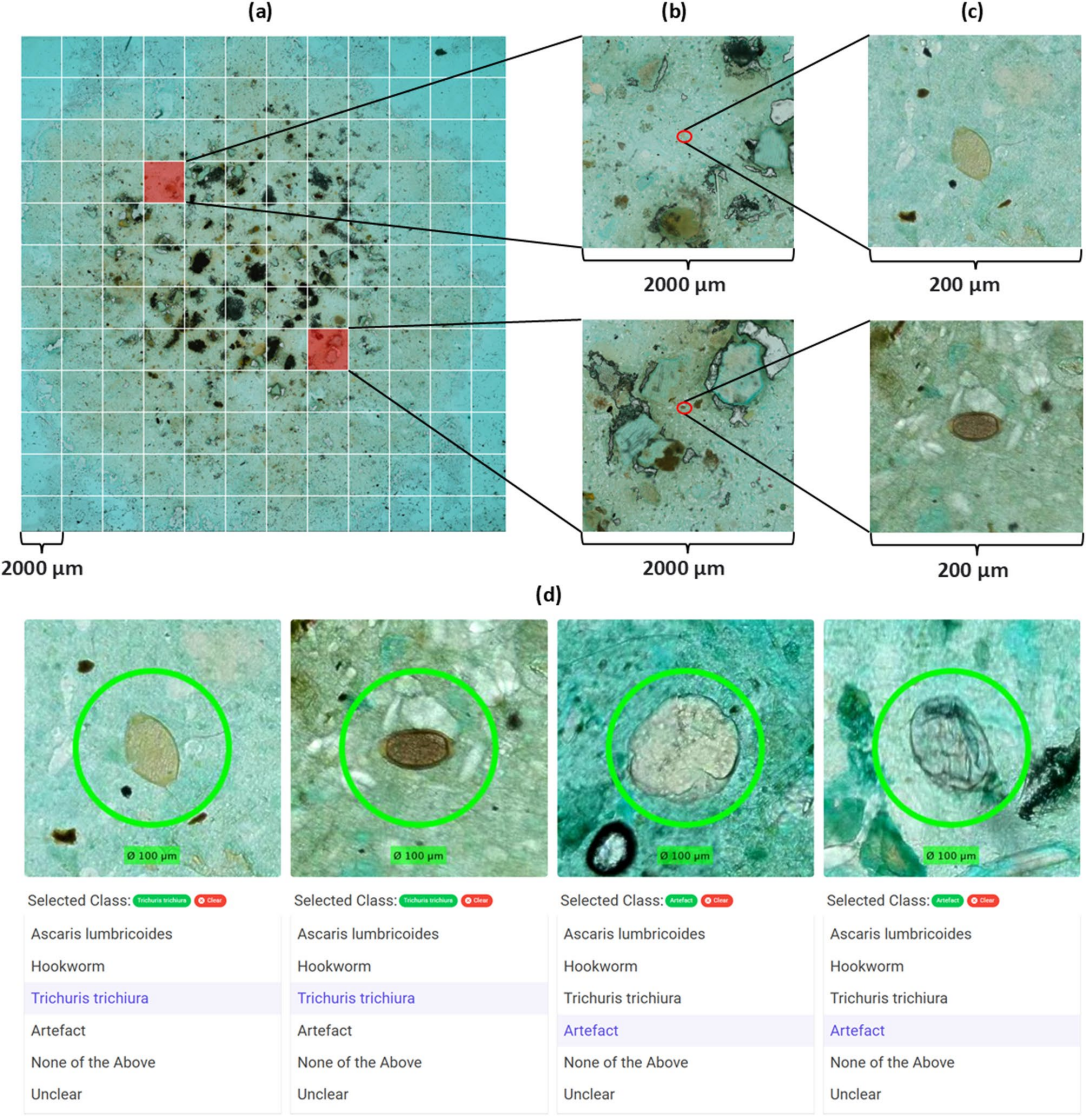
Visualization of findings from the same light-intensity infection smear, which was classified as negative by manual microscopy but positive by the expert-verified AI. (**a**) The whole Kato-Katz smear with a grid representing fields of view (colored red if positive) under a 10X objective; (**b**) the two fields of view with parasite eggs in the microscope; and (**c**) a close-up of the two parasite eggs (T. trichiura). (**d**) Visualization of the four objects detected by the DL algorithms as potential parasite eggs in the AI verificator tool.

Of the 122 smears classified as STH-positive according to the composite reference standard, 118 (96.7%) were classified as light intensity infections or negative by all the diagnostic methods. The remaining four smears were classified as follows: two *A. lumbricoides* as high intensity by all diagnostic methods, one hookworm as moderate intensity by the two digital methods and light intensity by manual microscopy and, one hookworm as high intensity by the two digital methods and light intensity by manual microscopy. Furthermore, 60 smears (*A. lumbricoides*: 3, *T. trichiura*: 34 and hookworm: 23) were unanimously identified as containing ≤4 eggs per Kato-Katz smear corresponding to < 100 eggs per gram (EPG). Of the 40 smears classified as negative by manual microscopy but positive according to the composite reference standard, 30 (75%) had ≤4 eggs detected by the other methods, an example of such a smear is shown in Fig. 1.

### Diagnostic accuracy of the three methods

The expert-verified AI had significantly higher sensitivity than manual microscopy for detecting *T. trichiura* (*p* < 0.001) and hookworm (*p* = 0.019). Similarly, the autonomous AI had significantly higher sensitivity for detecting *T. trichiura* (*p* < 0.001) than manual microscopy. Conversely, manual microscopy had significantly higher specificity than the autonomous AI for detecting *A. lumbricoides* (*p* = 0.016), *T. trichiura* (*p* = 0.001), and hookworm (*p* < 0.001), as well as higher specificity than the expert-verified AI for detecting hookworm (*p* = 0.001) (Table 2).

**Table 2:** Diagnostic accuracy of the different diagnostic methods compared to the composite reference standard.

Method	Manual microscopy		Autonomous AI		Expert-verified AI	
	Sensitivity, % (CI95%)	Specificity, % (CI95%)	Sensitivity, % (CI95%)	Specificity, % (CI95%)	Sensitivity, % (CI95%)	Specificity, % (CI95%)
Species						
*A. lumbricoides*	50.0 (11.8–88.2)	100 (99.5–100)	50.0 (11.8–88.2)	99.0(97.9–99.6)	100 (54.1–100)	99.3 (98.3–99.8)
*T. trichiura*	31.2 (16.1–50.0)	100 (99.5–100)	84.4 (67.2–94.7)	98.4 (97.1–99.2)	93.8 (79.2–99.2)	99.3 (98.3–99.8)
Hookworm	77.8 (67.8–85.9)	100 (99.4–100)	87.8 (79.2–93.7)	97.4 (95.8–98.5)	92.2 (84.6–96.8)	98.2 (96.8–99.1)

95% confidence interval (CI 95%).

The diagnostic accuracy with 95% confidence intervals (CI95%) of the digital methods was also calculated with the original DL-algorithms^14^, without the additional disintegrated hookworm detection algorithm. For the autonomous AI that resulted in a sensitivity of 55.6% (CI95% 44.7–66.0) and specificity of 98.4% (CI95% 97.0-99.2); and for the expert-verified AI a sensitivity of 61.1% (CI95% 50.3–71.2) and a specificity of 98.7% (CI95% 97.4–99.4). When the additional disintegrated hookworm DL algorithm was included, sensitivity significantly increased for both the autonomous AI (*p* < 0.001) and the expert-verified AI (*p* < 0.001). However, the detection of disintegrated hookworm eggs led to a significant decrease in specificity for the autonomous AI (*p* = 0.03) but not for the expert-verified AI (*p* = 0.25).

### Egg counts of the three methods in positive smears

**Fig. 2:**
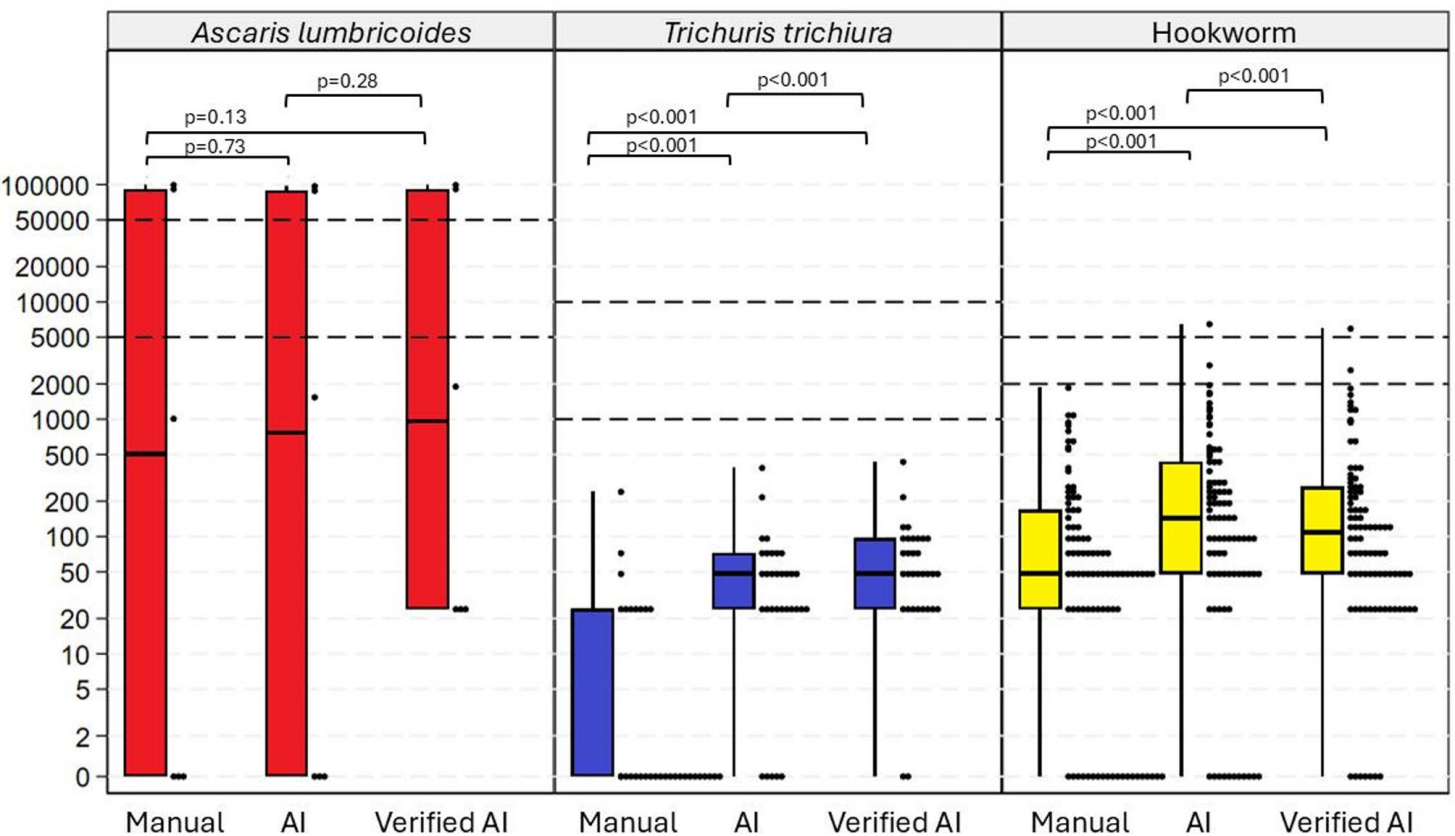
Egg count for the three diagnostic methods. The graph includes all smears that were positive according to the composite reference standard. The Y-axis represents eggs per gram. Cutoffs for the WHO’s definitions of light, moderate, and high-intensity infections have been marked with dashed lines for each species. *Two smears of A. lumbricoides were marked as uncountable in manual microscopy, for these smears the egg count from the expert-verified AI was used.

When comparing the egg counts of the positive smears according to the composite reference standard, the two digital methods had significantly higher egg counts than manual microscopy for *T. trichiura* and hookworms (*p* < 0.001 for both). When comparing the digital methods, the expert-verified AI yielded significantly higher egg counts for *T. trichiura* (*p* < 0.001) whereas the autonomous AI yielded significantly higher egg counts for hookworms (*p* < 0.001). Differences in *A. lumbricoides* egg counts were not significant between any of the diagnostic methods (Fig. 2).

## Discussion

This study compared the diagnostic accuracy of three methods for STH detection in Kato-Katz thick smears: manual microscopy, autonomous AI and expert-verified AI. The digital methods were based on an AI-method from our previous study which was further improved by the introduction of an additional DL-algorithm for the detection of partially disintegrated hookworm eggs and the AI-verificator tool for expert assessment of AI findings^14^. The vast majority (97%) of the positive smears were light-intensity infections, with only four being categorized as either moderate- or high-intensity infections by at least one diagnostic method.

The highest sensitivity for detection of all three STHs was achieved by the expert-verified AI (where an expert was shown findings in the AI-verificator tool). The expert-verified AI achieved higher sensitivity than the autonomous AI because the expert was shown objects the autonomous AI considered artifacts (confidence between 0.5 and 0.9) and reclassified them as positive. Manual microscopy had the lowest sensitivity for detection of all three STHs (tied with the autonomous AI for *A. lumbricoides*). Both digital methods identified more eggs in the positive smears than manual microscopy, which is consistent with the higher sensitivity of smear level analysis. The specificity for all diagnostic methods and species was above 97% with the manual microscopy having the highest specificity for all STHs followed by the expert-verified AI and then the autonomous AI. Since the composite reference assumed that findings in manual microscopy are correct, a noteworthy finding was that the autonomous AI was able to achieve a high specificity without relying on any human verification. When comparing the diagnostic accuracy of the methods, the main difference was in the sensitivity, where the expert-verified AI was superior to the other methods.

Our results align with those of other studies, where AI has shown high diagnostic performance for STHs at the parasite egg level^13,20,21^ and smear level^14,18,19^. One study that investigated *T. trichiura* showed that it is possible to identify more eggs in large fields-of-view with AI-assisted analysis than with manual microscopy^18^. Another study, where an AI-supported digital microscopy method was compared to manual microscopy, showed that the AI correctly identified more positive smears of *A. lumbricoides* whereas the amounts of *T. trichiura* and hookworms were similar^19^. In a previous report from our team, 10% of Kato-Katz smears classified as negative by manual microscopy and positive by a DL-algorithm contained manually verified parasite eggs in the digital smears, and the AI supported digital method generally identified more eggs than manual microscopy in positive smears^14^. This study, however, is the first to our knowledge that shows that expert-verified AI-analysis can increase the sensitivity for all STH species (with statistical significance for *T. trichiura* and hookworms) on a smear level in Kato-Katz thick smears compared to manual microscopy.

Hookworm disintegration is a well-known issue with the Kato-Katz technique, and it was hypothesized to be the reason for the relatively low sensitivity of hookworms in our previous study, as partially disintegrated hookworm eggs detected in digital smears were falsely classified as negative by the AI-method^10,14^. The improved disintegrated hookworm detection algorithm presented in the current study increased the sensitivity for hookworms for both the autonomous AI (from 55.8 to 87.8%) and the expert-verified AI (from 61.1 to 92.2%). The sensitivity improvements demonstrate the benefit of using DL-algorithms that can identify parasite eggs with variable morphology, such as partially disintegrated hookworm eggs.

A limitation of our study is that the composite reference standard is not a true gold standard and was based on visual assessments by two experts (FK and KO) in the physical and digital Kato-Katz thick smears, with no inclusion of any alternative methods. Therefore, the composite reference standard likely contains smears falsely classified as negative due to the inherent weaknesses of the manual Kato-Katz technique. The challenge of identifying helminth eggs in smears with light intensity infections is illustrated in Figs. 1 and 75% of false negative smears in manual microscopy had four or fewer eggs detected by both the autonomous and the expert-verified AI. As a result, the sensitivities of the three methods are likely overestimated, making comparisons with other sample preparation techniques or molecular methods such as FLOTAC or PCR challenging. However, since manually verified eggs are generally considered to be highly specific^22,23^, the number of false positive smears in our composite reference standard can be assumed to be low, allowing for reliable comparisons between the three diagnostic methods evaluated in this study. To account for potential misclicks in the AI-verificator, findings in the digital smear had to be verified by two experts (FK and KO) whereas only a single expert (FK) performed manual microscopy of the physical smears.

A limitation is that only one expert (FK) performed the manual microscopy, because of the short timespan available to perform microscopy of the physical smears before hookworm disintegration. Also, the results for expert-verified AI represent a single user, since we chose to minimize the inter-observer variability by having the same expert that performed manual microscopy to use the AI-verificator tool.

Another limitation is the high number of samples that were excluded (*n* = 261). The two main reasons for exclusion were the 181 inadequate samples (for example because of sand contamination, the stool containing excessive oil or vegetable cells or the stool consistency being too loose or hard) and the 60 smears which had no available scans. The smears with missing scans were excluded prior to analysis (due to reasons such as no researcher operating the scanner or issues with the uploads of the digital smears) and should therefore not introduce any bias in the comparison of the three diagnostic methods. This is supported by the fact that the STH prevalence in the 60 excluded smears was similar to that observed in the 704 smears included in the main analysis. A further limitation of the study is that manual microscopy was performed prior to scanning of the smears; therefore, hookworm disintegration and clearing of the sample (improved contrast against the background) potentially revealing eggs of other species may have occurred before scanning. Clearing may explain why more positive smears of *T. trichiura* were identified with the two digital methods^11^. Delaying the manual microscopy reading, including multiple manual and digital readings, or randomizing the order in the workflow may have mitigated this limitation, and should be considered in future studies. Another limitation of the study is the small number of smears with *A. lumbricoides* and *T. trichiura* (6 and 32, respectively), and the fact that half of the *Ascaris* smears contained only a single verified egg and all *T. trichiura* smears were light intensity infections, with only four smears containing more than four eggs per smear according to any diagnostic method. This could explain the low sensitivity of manual microscopy but also shows the strength of the expert-verified AI for detection of light intensity STH infections.

With the global decline of STHs, diagnostic methods with high accuracy are critically needed to guide programmatic policy decisions and test-and-treat approaches^7,8^. The results of our study indicate that implementing digital microscopy and expert-verified AI may improve the diagnosis of STHs in populations mainly harboring light intensity infections. According to the WHO target product profile for STH diagnostics, a sensitivity of above 77% and a specificity above 97% is considered ideal, and our proposed expert-verified AI fulfills this criteria for all STH-species in the current study against the composite reference standard^8^. Performing the analysis with the expert-verified AI locally would take 11–16 min (scanning 5–10, AI-analysis 5 and expert-verification 1 min). Since only approximately one minute is expert hands on, the method could provide rapid diagnosis and reduce the workload of local experts. To further investigate the diagnostic accuracy of AI-supported digital microscopy for Kato-Katz thick smears it would be important to compare the method with molecular methods and other advanced microscopy methods, such as FLOTAC or McMaster. Furthermore, research on the cost efficiency of AI-supported digital microscopy would be warranted.

## Conclusion

This study presents a method that combines a portable whole-slide scanner with DL-based AI for STH detection, implemented in a real-world primary healthcare laboratory setting. The expert-verified AI correctly identified more Kato-Katz thick smears as positive than the manual microscopy with a majority being light intensity infections, with statistically significant improvements in the detection of *T. trichiura* and hookworms. The sensitivity of expert-verified AI was higher than that of both manual microscopy and autonomous AI while maintaining high specificity for all STHs.

## Methods

### Study design

**Fig. 3:**
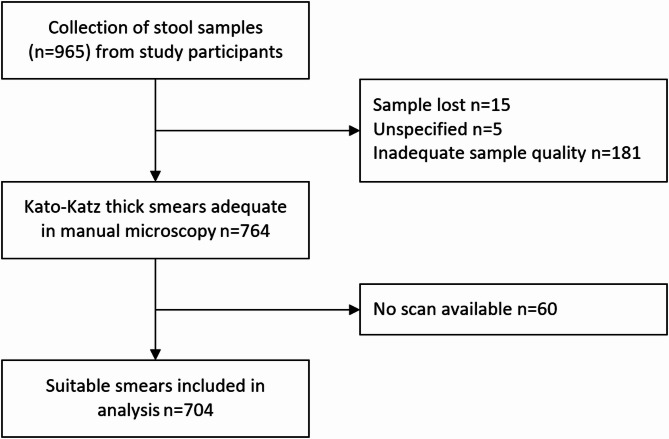
Standard for Reporting Diagnostic Accuracy (STARD) Flow Chart of the Kato-Katz samples collected.

The diagnostic accuracy of three methods for detection of STHs in Kato-Katz thick smears was compared to a composite reference standard. The methods were: manual microscopy, an autonomous AI-based digital method and an expert-verified AI. To evaluate these three methods, 965 stool samples were collected from school children in Kwale County, Kenya. The study was conducted at the Kinondo Kwetu Hospital (https://www.kinondokwetuhospital.com), a primary health care hospital, owned and supported by a trust fund (Kinondo Kwetu Trust Fund). The study flow is presented in accordance with the Standards for Reporting of Diagnostic Accuracy Studies (STARD)-guidelines in Fig. 3^24^.

### Overview of the three diagnostic methods

**Fig. 4:**
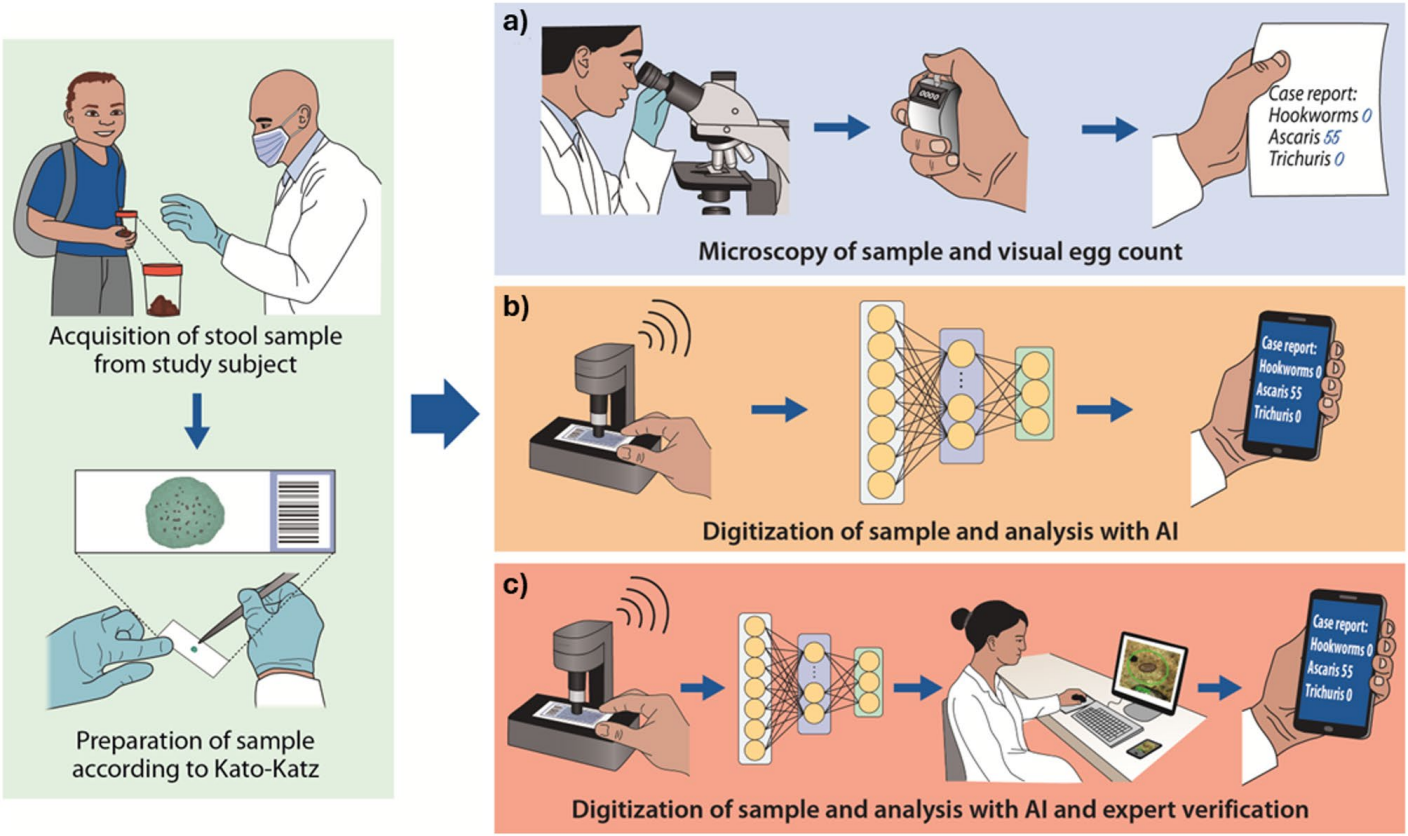
Workflow for the different methods. On the left: Collection and preparation of smears. On the right: Steps in the three diagnostic methods: (**a**) Manual microscopy, (**b**) Autonomous artificial intelligence (AI), and (**c**) The expert-verified AI.

Each Kato-Katz thick smear was analyzed individually using the three diagnostic methods. All methods shared the same sample collection and preparation process (Fig. 4), and the two digital methods also shared the scanning procedure and parts of the analysis procedure (Fig. 4b and c), described in detail in the following paragraphs.

### Collection of stool samples and Preparation of Kato-Katz Thick smears

Stool samples were collected from school children (age 5–16) either at their homes or at the Kinondo Kwetu Hospital (Kwale County, Kenya) between March 2020 and April 2021. A total of 965 stool samples were collected from 898 participants. A single Kato-Katz thick smear was prepared from each stool sample. For patients with an initial positive stool sample, a second sample was collected four days after treatment initiation to assess treatment outcome, resulting in more samples than participants in the study. Smears were excluded if they were inadequate. The main reasons for a smear being deemed inadequate were as follows: contamination with sand of the stool, oil or high amount of vegetable cells in the stool (leading to obscured parasite eggs), poor stool consistency (to hard or diarrhea) or fragmented filtrate causing empty or dense areas in the smear. For the samples in which microscopy analysis failed a second sample was collected when possible. The fecal samples were transported to Kinondo Kwetu Hospital, where they were assigned a study code and prepared by trained laboratory technicians using the Kato-Katz staining technique^10^.

### Manual microscopy

The smears were analyzed using a manual light microscope (CX23; Olympus, Tokyo, Japan) by an expert (FK) within 5 min after preparation to minimize hookworm disintegration. The entire cellophane-covered area was examined at 10X or 40X magnification with numerical apertures (NA) of 0.25 and 0.65 respectively. Smear quality was monitored by the expert (FK) performing the microscopy. Additionally, the technician conducting scans (MM) assessed the physical and digital smear quality. When issues arose, the sample quality was discussed and smears were re-prepared from the original stool sample if this could resolve the issue; otherwise, an attempt to collect a new stool sample was made. Furthermore, digital smear quality was assessed continuously by off-site researchers. The parasite eggs were counted for the respective STH species detected.

### Digitization of the smears

After manual microscopy, the smears were digitized using a portable whole-slide scanner (Ocus, Grundium, Finland) equipped with a 6-megapixel image sensor and a 20X objective (NA 0.40), producing a digital whole slide image with a pixel size of 0.48 μm. Before scanning a smear, the coarse focus was manually adjusted, and the built-in autofocus was subsequently used for fine-tuning. The entire cellophane-covered area was scanned, and the digital smears were initially saved in Tagged Image File Format (TIFF). The scanning time was 5–10 min. The smears were then converted into JPEG-compressed tiles sized at 512 × 512 pixels, with a quality of 70% before being uploaded to the image management platform (Aiforia Hub, Aiforia Technologies, Helsinki, Finland) using a mobile network (Diani Networks Limited, Kenya). On the image management platform, the digital smears were converted into JPEG-compressed tile maps with a pyramid zoom level structure. Afterwards, the digital smears were downloaded from the image management platform for further processing in MATLAB (MathWorks Inc, Natick, MA, USA). The uploading and downloading time was in total 10–20 min per sample with mobile network. No digital scans were available for some smears (*n* = 60). Out of these 60, 40 were not scanned (for example because there was no researcher available who could operate the scanner at the time), 13 were scanned and not uploaded (for example because the scanner had no memory left and could not save the scan) and for the remaining seven no explanation was available.

### AI-model for image analysis

Training and inference were performed on a PC workstation equipped with an Intel Xeon E3-1241 v3 CPU, an NVIDIA GeForce GTX1660 Super GPU, and 32GB of RAM, running MathWorks MATLAB R2022b on a Microsoft Windows 10 operating system. The complete analysis with the AI-model took about 5 min per sample.

### Development of the DL-algorithms

The AI-model consisted of three sequential DL-algorithms. The first two formed a complete model in our previous study and were not retrained or modified within this study, and the third was trained in this study to improve the original model^14^. The training data used for the development of DL-algorithms in the previous study was gathered from 388 Kato-Katz thick smears, and 15,058 training regions that measured 512 × 512 pixels were annotated through AI-assisted manual annotation (where earlier DL-algorithms were used to identify potential parasite eggs, which were manually classified to train the next iteration of the DL-algorithms). The training regions used contained: *A. lumbricoides* (*n* = 2,299), *T. trichiura* (*n* = 2,727), hookworms (*n* = 552) and artefacts (*n* = 9,480). The two DL-algorithms operated sequentially. The first (detector-algorithm) was trained to detect suspicious objects, and the second (classifier-algorithm) to classify objects into one of four categories: *A. lumbricoides*, *T. trichiura*, hookworm or artefact (i.e., debris or other non-STH objects). Further details on the two initial DL-algorithms are described in our previous study^14^.

**Fig. 5:**
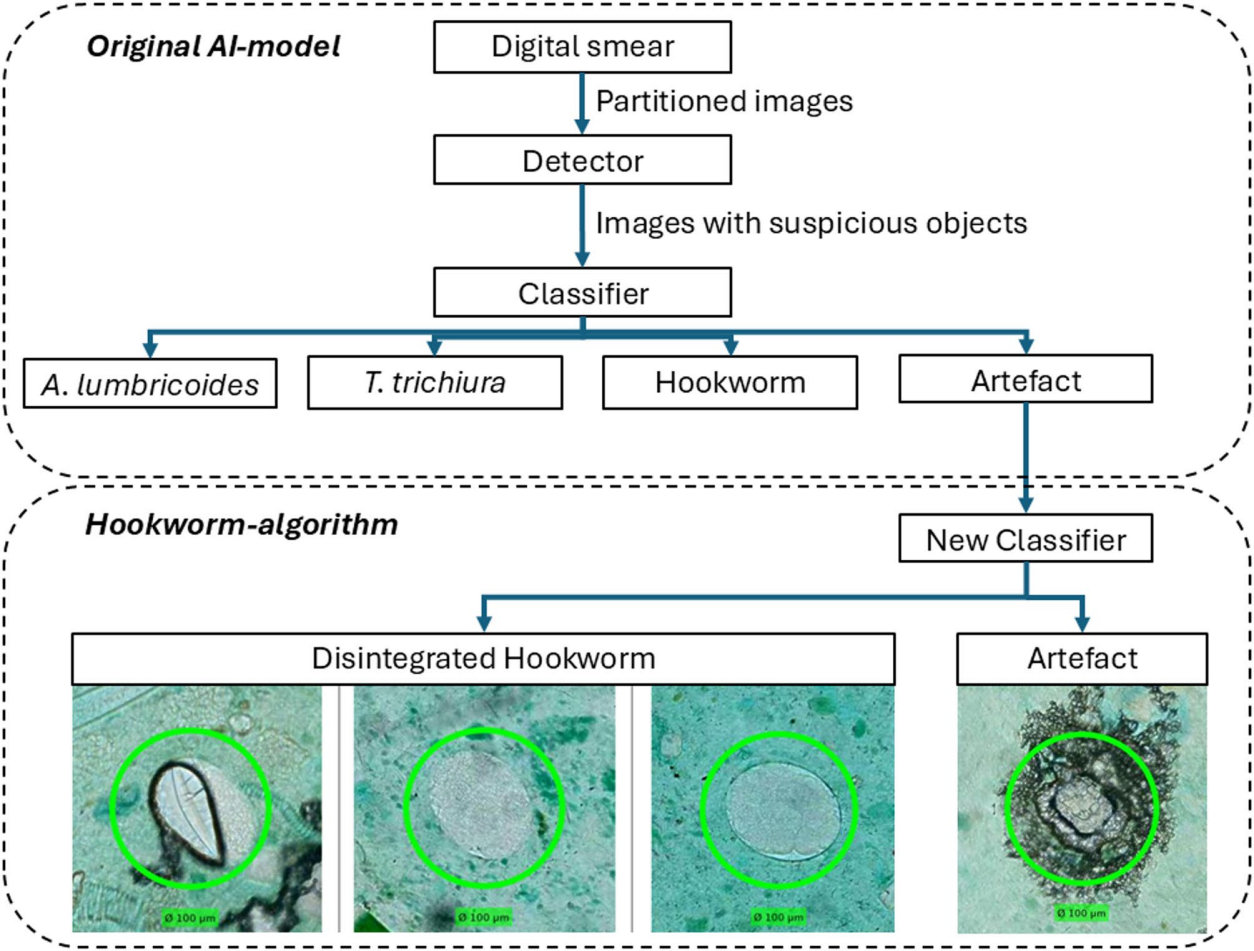
Visualizing the Original AI-model and the additional disintegrated hookworm detection algorithm. First step: partitioned images from digital smears are passed through the detector-algorithm. Second step: classification of the parasite egg candidates into soil-transmitted helminth and artefact categories. Third step: classification of artefacts into disintegrated hookworm eggs and “artefacts” and examples of objects classified into each group.

The third DL-algorithm that was trained in this study was based on the ResNet50 architecture^25^ and was trained using transfer learning to identify disintegrated hookworm eggs^26^. The training data were gathered from the image regions (150 × 150 pixels) classified as artifacts by the first two DL-algorithms in smears from the previous study^14^. The annotations were made using AI-assisted manual annotation by three researchers (JvB, AS and FK), and the consensus label was used for training. The final training dataset contained 777 disintegrated hookworms and 991 objects with hookworm-resembling morphology. The training data were randomly partitioned into five subsets. K-fold cross-validation was used to train five convolutional neural networks (ResNet50), where each was trained using four different subsets as the training set (80%) and the remaining subset as the validation set (20%). The training images were augmented with multiple randomized transformations, including scale manipulation (± 10%), rotation (0-360°), XY shear (± 15°), XY reflections and XY translations (± 15 pixels) and color augmentations with saturation offset (0.1), brightness offset (0.2), hue offset (0.05), and contrast scale factor (0.2). Each network was trained for a maximum of 100 epochs with a minibatch size of 32. A stochastic gradient descent solver with a momentum of 0.9 was deployed, with an initial learning rate of 0.003, and a 50% reduction in the learning rate every 10 epochs. Validation was performed after each epoch, and the training was stopped early if the validation loss did not improve within 10 epochs. The network with the best validation loss from each training session was selected as the final output network. All five convolutional neural networks were combined into one DL-algorithm, and their output confidence scores were averaged to produce a single confidence score. The finalized DL-algorithm was then used to classify the objects labeled as artifacts by the previous DL-algorithm as either a disintegrated hookworm or “artefact” (Fig. 5).

### Application of DL-algorithms on digital smears

The first step of the digital analysis with the AI-model was to create small regions of 512 × 512 pixels (246 × 246 μm) with an overlap of 128 pixels (61 μm) with adjacent regions to cover the entire digital smear. Each partitioned region was analyzed using the detector-algorithm which identifies suspicious objects. When an object was detected in multiple overlapping bounding boxes, the box with the highest confidence (i.e. the probability score) was selected and the other boxes were excluded.

Detected objects were then resized to 150 × 150 pixels (72 × 72 μm) and forwarded to the classifier algorithm, where confidence scores for each class were generated. In the autonomous AI method, objects with an egg parasite confidence of > 0.9 were classified as parasite eggs. In the expert-verified AI method, objects with an egg confidence of > 0.5 or an artifact confidence score of < 0.95 were uploaded to the AI verification app for manual verification.

All objects classified as artefacts by the classifier-algorithm were passed through the additional disintegrated hookworm detection algorithm to identify disintegrated hookworm eggs. The improved hookworm-algorithm classifies each image as either a disintegrated hookworm egg or “artefact”. Each object classified as a disintegrated hookworm egg with a confidence level of > 0.99 was considered positive in the autonomous AI-method. For the expert-verified AI, objects with a confidence of > 0.96 were uploaded to the AI-verificator tool.

Some objects included in the expert verification were uploaded twice as they fulfilled the criteria from both the original classifier and the disintegrated hookworm detection algorithm. These were objects which the classifier-algorithm considered an egg with a confidence of 0.5–0.9; thus, they were both classified as an artefact (and therefore analyzed by the hookworm-algorithm) and included in expert verification because of an egg confidence > 0.5. The final verification label was used for these objects.

### Manual verification of AI-findings

The AI-verificator is a web application tool developed to enable experts to visualize and verify AI findings. An interactive user interface was created using components from an open-source library (Mud Blazor UI, MudBlazor). Additionally, Docker (Docker Inc, USA) and Azure DevOps (Microsoft, USA) were used in an iterative process to improve the tool based on expert feedback. Serverless functions and Web APIs were developed to enable secure data import and export. Role-based access was implemented to ensure secure data management using MS Azure Entra ID and additional Azure resources such as Virtual Private Network, Azure Cosmos DB and Azure Storage (Microsoft, USA).

**Fig. 6:**
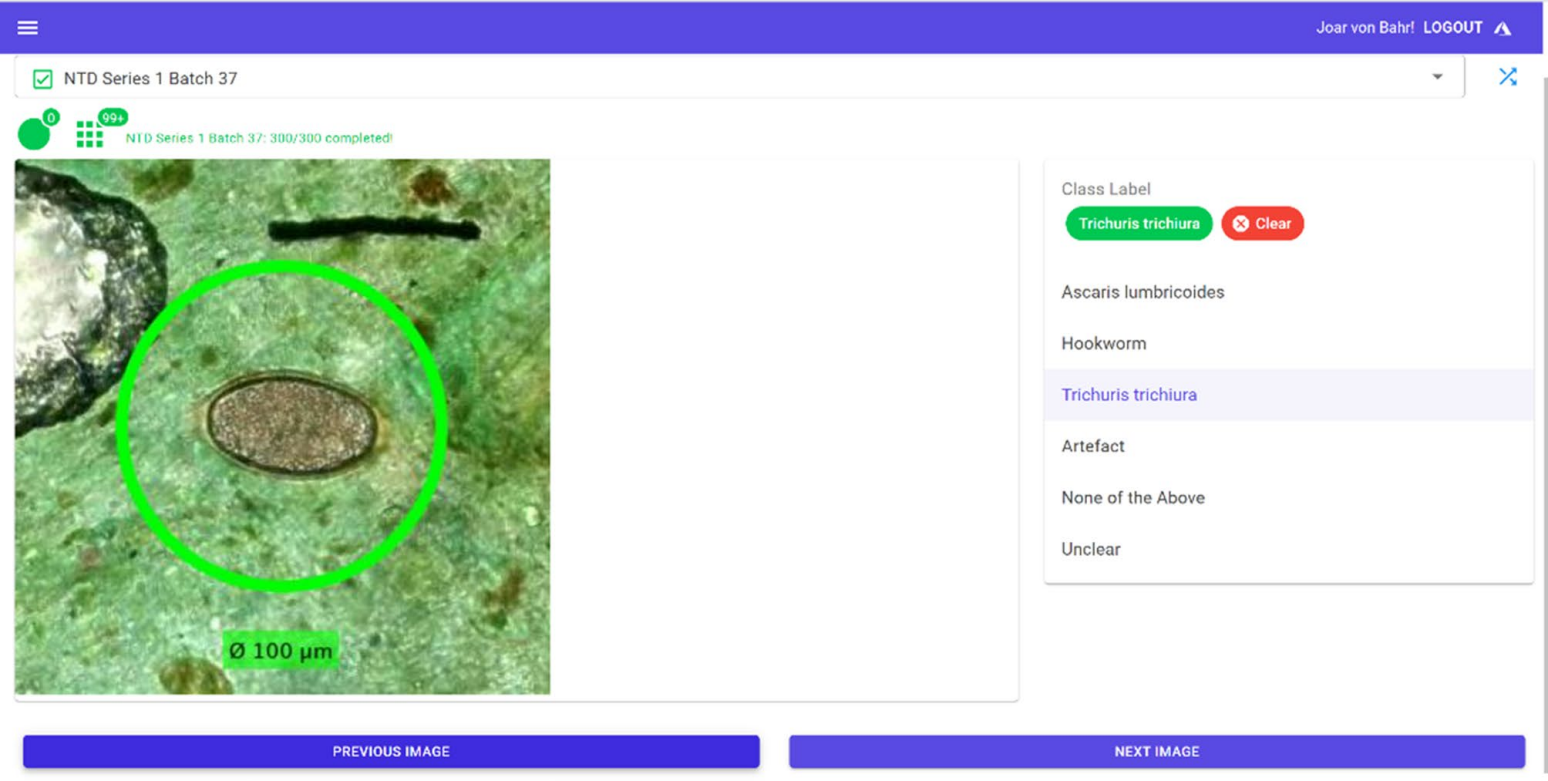
The view of the AI-verificator tool in single object verification mode.

The AI-verificator enables the classification of objects performed through multiple microtasks. The options included: *A. lumbricoides*, *T. trichiura*, hookworm, none of the above (e.g. parasite eggs not included in the study), artefact or unclear (e.g. not determinable because of poor focus, object obscuration, unusual characteristics etc.). The images are visualized either as a single image (Fig. 6) or as a panel of multiple images (Fig. 1d). The results obtained at the object level were used to generate a smear-level diagnosis. The mean time to verify a suspicious object was on average 5 s making the average time to verify a smear approximately 1 min. To avoid inter-observer variability between the expert-verified AI and the manual microscopy, the same expert (FK) performed the verification in the AI-verificator tool and manual microscopy of the smears.

### Composite reference standard

The three methods were compared with a composite reference standard that included manually and digitally verified positive Kato-Katz thick smears. The composite reference standard assumes that the manually verified findings were correct and were consequently defined as true positives. Previous studies have adopted similar approaches assuming specificities for STHs of close to or at 100% for multiple microscopy methods including Kato-Katz thick smears^22,23^.

The samples were considered to contain manually verified eggs and thus positive if one of two criteria was fulfilled: First, the expert (FK) who performed the manual microscopy identified eggs in the physical smear. Second, if a suspicious object presented in the AI verification app was identified independently as an egg by two experts (FK and KO) in the digital smear. The remaining slides, without any eggs identified in either the physical or digital smears, were considered negative.

### Statistical analysis

The necessary sample size for the study was based on the estimations in our previous study^14^. Based on these calculations, 692 and 173 samples were required for sensitivity and specificity, respectively^14^. Data from each sample were entered into a spreadsheet (Microsoft Excel, Microsoft, Redmond, WA, USA). The analyses were performed using general-purpose statistical software (Stata, version 18.0, College Station, TX, USA) and the metrics evaluated for diagnostic accuracy were specificity and sensitivity. Sensitivity and specificity were calculated separately for each species. Statistical estimates of diagnostic accuracy were reported with a CI95%. The level of statistical significance was set at 0.05 for all analyses. Further, the positive and negative predictive values were calculated for each method and are available in the Supporting Information. To evaluate the statistical significance between the different diagnostic methods, McNemar x^2^ was applied as proposed by Trajman and Luiz^27^. To approximate the EPG of stool it was estimated that each sample contained 41.7 mg stool, rendering a factor of 24 (24 × 41.7 mg ≈ 1 g)^9^. P-values for comparison of egg counts in the positive smears between the diagnostic methods were calculated using the Wilcoxon signed-rank test, with the exact probabilities used. This was done since the paired egg-counts were non-normally distributed and the number of positive smears was low for *A. lumbricoides* (*n* = 6).

## Electronic supplementary material

Below is the link to the electronic supplementary material.

Supplementary Material 1

## Data Availability

All data required to evaluate the conclusions in the article are included in the manuscript and/or the supplementary material. Additional data are available on request from the Data Access Committee (FIMM-DAC) at Institute for Molecular Medicine Finland (FIMM), University of Helsinki, Helsinki, Finland; fimm-dac@helsinki.fi. Further requests for sharing of deidentified data (digitized samples) will be considered by the FIMM-DAC abiding the following principles: data will be securely stored with appropriate documentation and not disposed into publicly accessible domains or otherwise shared without explicit permission from the FIMM-DAC, and data are only used with the aim to generate data for the public good.
